# Fatal complications in a young adult with large Ewing sarcoma of the chest wall

**DOI:** 10.1016/j.radcr.2026.09.024

**Published:** 2026-10-05

**Authors:** Arianna Smith, Andrew Gaetano, Elizabeth Sager, Sohail Hussaini, Emad Allam

**Affiliations:** Loyola University Medical Center, Loyola University Chicago, 2160 S 1st Ave, Maywood, IL 60153, USA

**Keywords:** Ewing sarcoma, Ewing sarcoma of chest wall, Hydropneumothorax, Osseous metastasis, Pathologic fracture

## Abstract

Ewing sarcoma is an aggressive bone and soft tissue malignancy arising predominantly in children and young adults, with a peak incidence at 15 years of age with a slight male predominance. The ribs are a recognized primary site of Ewing sarcoma, accounting for approximately 10% of cases. Tumors arising from the ribs/chest wall, often called Askin tumors, are the most common primary malignant chest wall tumor in children and young adults. These tumors are highly aggressive, often presenting with early micrometastatic disease and requiring prompt multimodal therapy.

We present the case of a 20-year-old female with an exceptionally large Ewing sarcoma of the anterior chest wall, likely originating from the rib, who was lost to follow-up after initial presentation 20 months earlier. On re-presentation, imaging demonstrated extensive progression of disease with invasion of the breast, lung, and chest wall, as well as widespread metastatic disease including vertebral involvement with pathologic compression fractures and spinal cord compression.

Despite initiation of multimodal therapy with chemoradiation, her clinical course was complicated by pleural effusions, hydropneumothorax, and progressive tumor necrosis. She ultimately developed acute respiratory distress syndrome (ARDS) following palliative surgical resection and passed away from her disease approximately three years after initial presentation. This case highlights the aggressive natural history of a chest wall Ewing sarcoma, and the role of imaging in identifying disease extent, complications, and progression.

## Introduction

Ewing sarcoma is an aggressive malignancy of likely mesenchymal origin that primarily arises in bone and soft tissue. Ewing sarcoma is most common in children and adolescents, with a peak incidence of 15 years of age [1,2]. The vast majority of Ewing sarcomas originate from bone (80%); however, about 20% of cases originate in the soft tissue as extraskeletal Ewing sarcoma (EES) [1]. Osseous lesions most commonly arise in the lower extremities (45%), pelvis (20%), and upper extremities (13%). Ribs and other axial skeleton sites account for approximately 13% of cases [3].

Ewing sarcoma is genetically associated with a characteristic t(11;22)(q24;q12) chromosomal translocation, which is present in 85–90% of cases [[3], [4]]. Histological examination reveals small, undifferentiated round blue cells with prominent nuclei and scant cytoplasm [1,3]. Imaging features can often vary based on tumor location; however, the most characteristic radiographic presentation is permeative destruction of long bones and interrupted periosteal new bone formation leading to an aggressive “moth-eaten” appearance [6]. The early presenting clinical features of Ewing sarcoma are nonspecific and occult metastasis is frequent, which leads to a significant diagnostic challenge [1]. Metastasis at the time of presentation is highly correlated with patient outcomes, with five-year survival rates reduced from 70% in those with localized disease to less than 30% with metastatic disease [5]. Standard of care treatment involves a multimodal treatment approach combining chemotherapy, tumor resection/reduction, and radiotherapy given the aggressive and high metastatic potential of the disease [1,6].

This case demonstrates an uncommon presentation of Ewing sarcoma, arising from the chest wall. It highlights the risks of rapid progression of disease with delayed presentation and treatment of a large, metastatic Ewing sarcoma and the importance of prompt clinical follow-up.

## Case presentation

### Clinical presentation

An 18-year-old female presented with a palpable right breast mass and underwent lumpectomy at an outside institution with presumed benign etiology. Histopathologic evaluation revealed a malignant small round blue cell tumor consistent with Ewing sarcoma, confirmed by immunohistochemical staining and EWSR1 gene rearrangement. Surgical margins were positive.

### Initial management and disease progression

Following initial resection, the patient did not undergo staging studies or standard adjuvant therapy. Over the subsequent 1 year and 6 months, she pursued alternative treatments including high-dose vitamin C, hyperbaric oxygen therapy, and low-dose chemotherapy.

Approximately 20 months after initial presentation, she re-presented to a tertiary care center with a markedly enlarged right breast mass measuring approximately 21 by 15 cm, fixed to the chest wall with overlying skin changes. The patient continued to seek alternative treatments.

### Progressive disease and treatment

Approximately 2 years after initial diagnosis, imaging demonstrated extensive disease. CT showed a large right breast and axillary mass extending into the lung with rib erosion and pleural effusion (Fig. 1). MRI confirmed a multilobulated, heterogeneously enhancing mass with chest wall invasion and mediastinal abutment (Fig. 2). Concurrent spinal metastases were identified with multiple compression/burst fractures and cord compression at T9 (Fig. 3), correlating with new lower extremity weakness. She was admitted emergently and treated with spinal radiation (30 Gy in 10 fractions) and systemic chemotherapy for cord compression, followed by interval-compressed multi-agent systemic chemotherapy with the VDC/IE backbone (vincristine, doxorubicin, and cyclophosphamide alternating with ifosfamide and etoposide). Despite initial therapy, interval disease progression prompted escalation to salvage chemotherapy with vincristine, irinotecan, and temozolomide. She also received consolidative and palliative radiation therapy to sites of disease, including approximately 60 Gy to the right chest wall, 15 Gy to the right hemithorax, and additional targeted radiation (up to 70 Gy) to recurrent chest wall and axillary lesions.

**Fig. 1:**
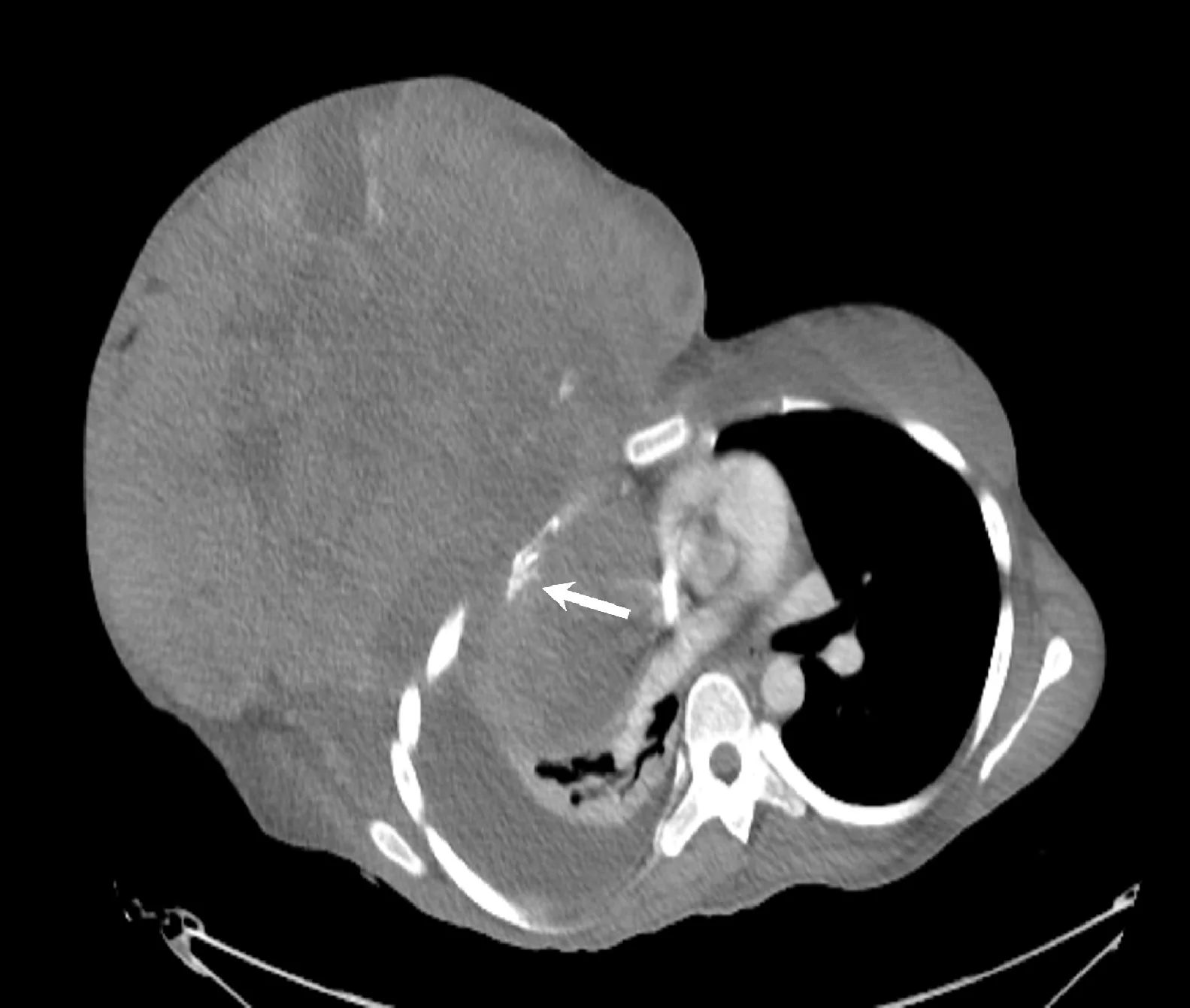
Axial contrast-enhanced CT image (soft tissue window) shows a large lesion in the right breast and axilla, extending into the right lung. There is erosion of the right anterior third rib (arrow). There is a moderate layering right pleural effusion with associated atelectasis.

**Fig. 2:**
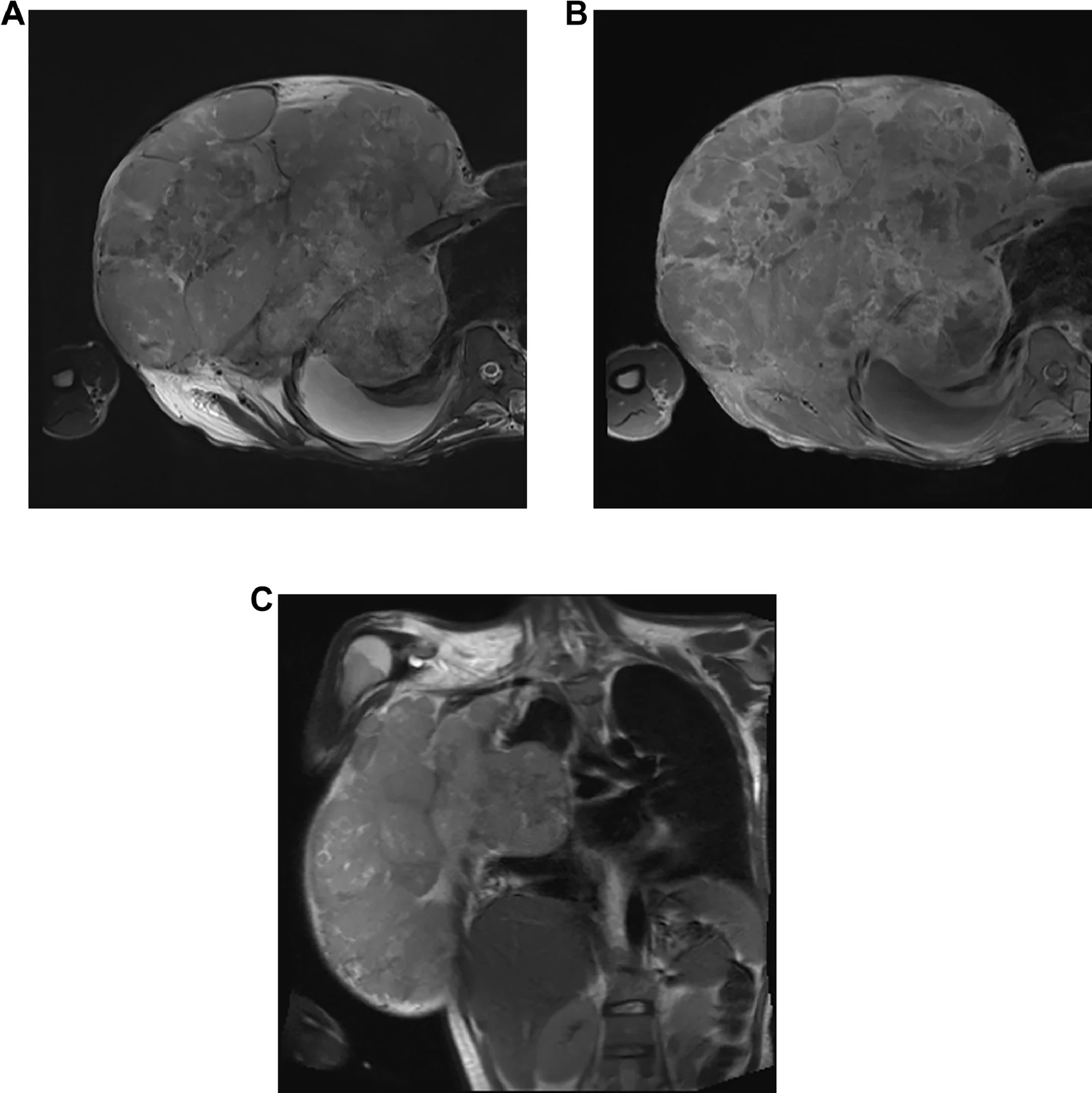
(**A**) Axial T2 FS (fat saturated) MRI image shows a large multilobulated lesion in the right breast and axilla, extending into the right lung. There is involvement/encasement of multiple right ribs. There is a moderate layering right pleural effusion. (B) Axial T1 FS post-contrast MRI image shows heterogeneous enhancement of the lesion. (C) Coronal HASTE MRI image shows a large lesion in the right breast, extending into the right lung and abutting the mediastinum.

**Fig. 3:**
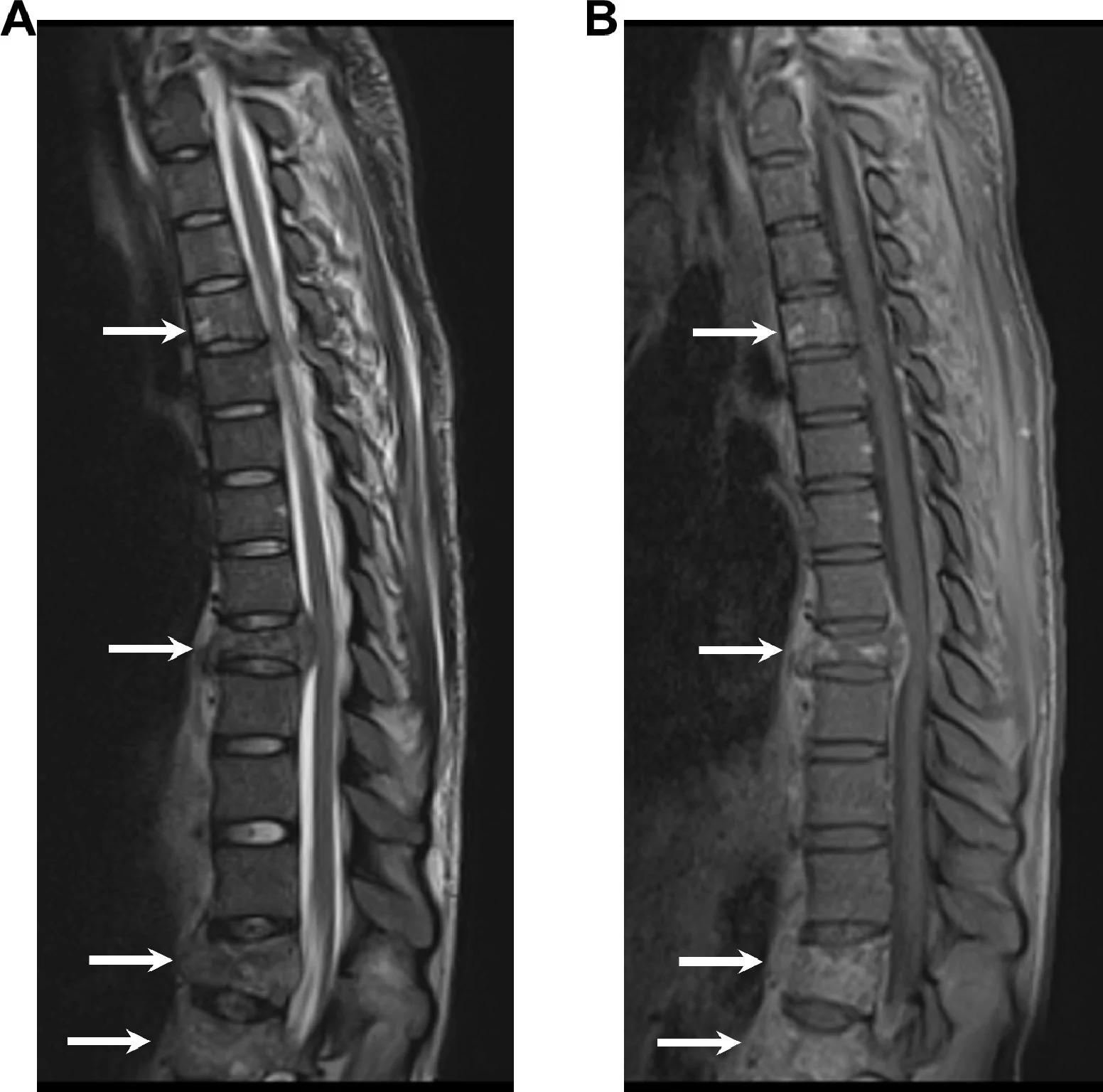
(**A**) Sagittal T2 FS MRI image shows pathologic compression/burst fractures of T4, T9, L1, and L2 (arrows). There is retropulsion at T9 with cord compression and mild cord signal abnormality. (B) Sagittal T1 FS post-contrast MRI image shows pathologic compression/burst fractures of T4, T9, L1, and L2 with underlying enhancing vertebral lesions (arrows).

Follow-up PET/CT at approximately 3 months after chemoradiation showed decreased tumor size but persistent fluorodeoxyglucose (FDG) avidity (Fig. 4**).** Approximately 9 months into treatment, there was further response of the primary lesion but new FDG-avid disease in the left breast (Fig. 5). Despite additional chemotherapy and radiation, disease progression continued over the next 6–9 months and targeted therapy with pazopanib was initiated.

**Fig. 4:**
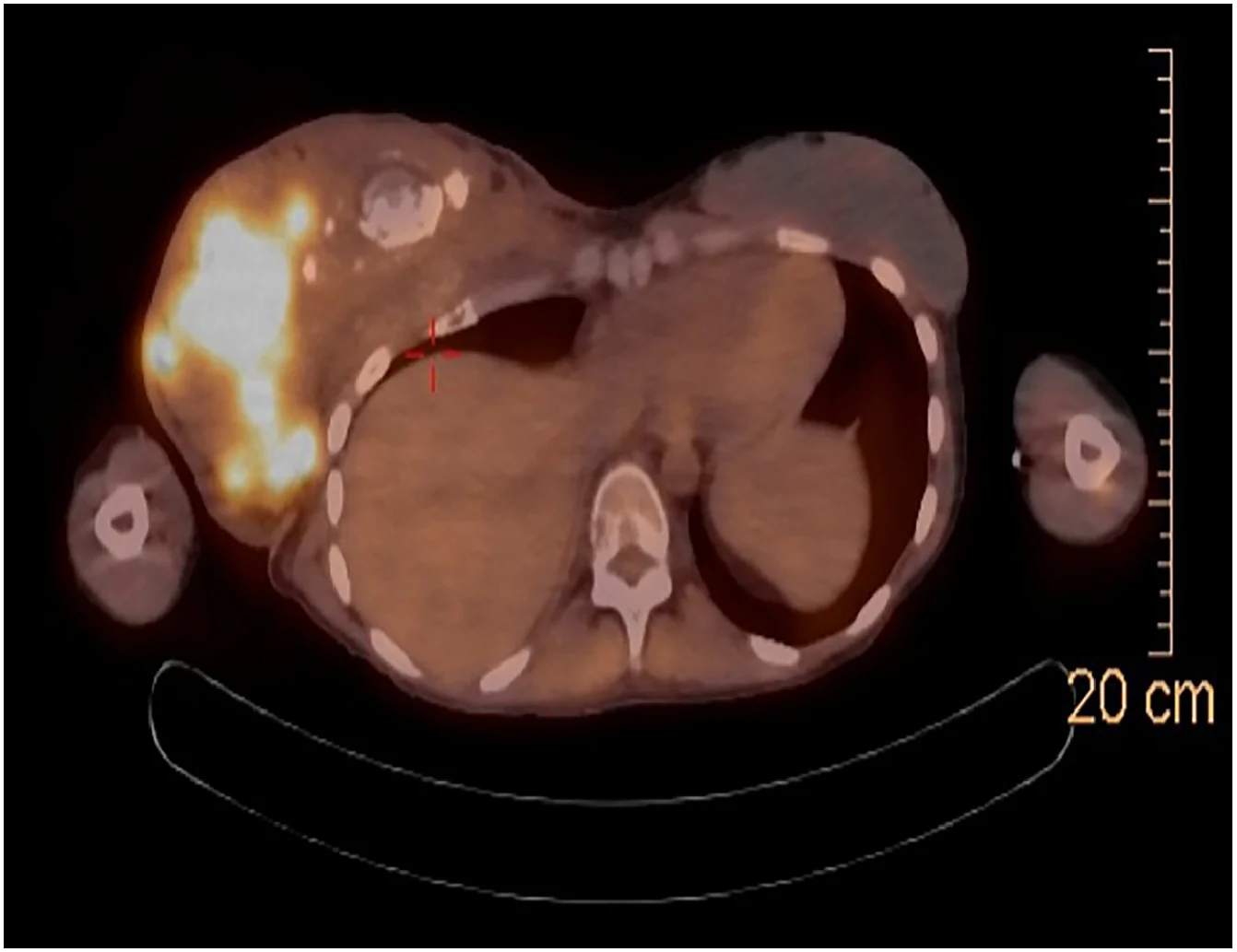
PET/CT obtained 3 months after the prior imaging shows decreased size of the right breast mass following treatment. There is intense FDG avidity in the outer aspect and interval calcifications in the inner aspect of the right breast on this image. The lungs are incompletely imaged.

**Fig. 5:**
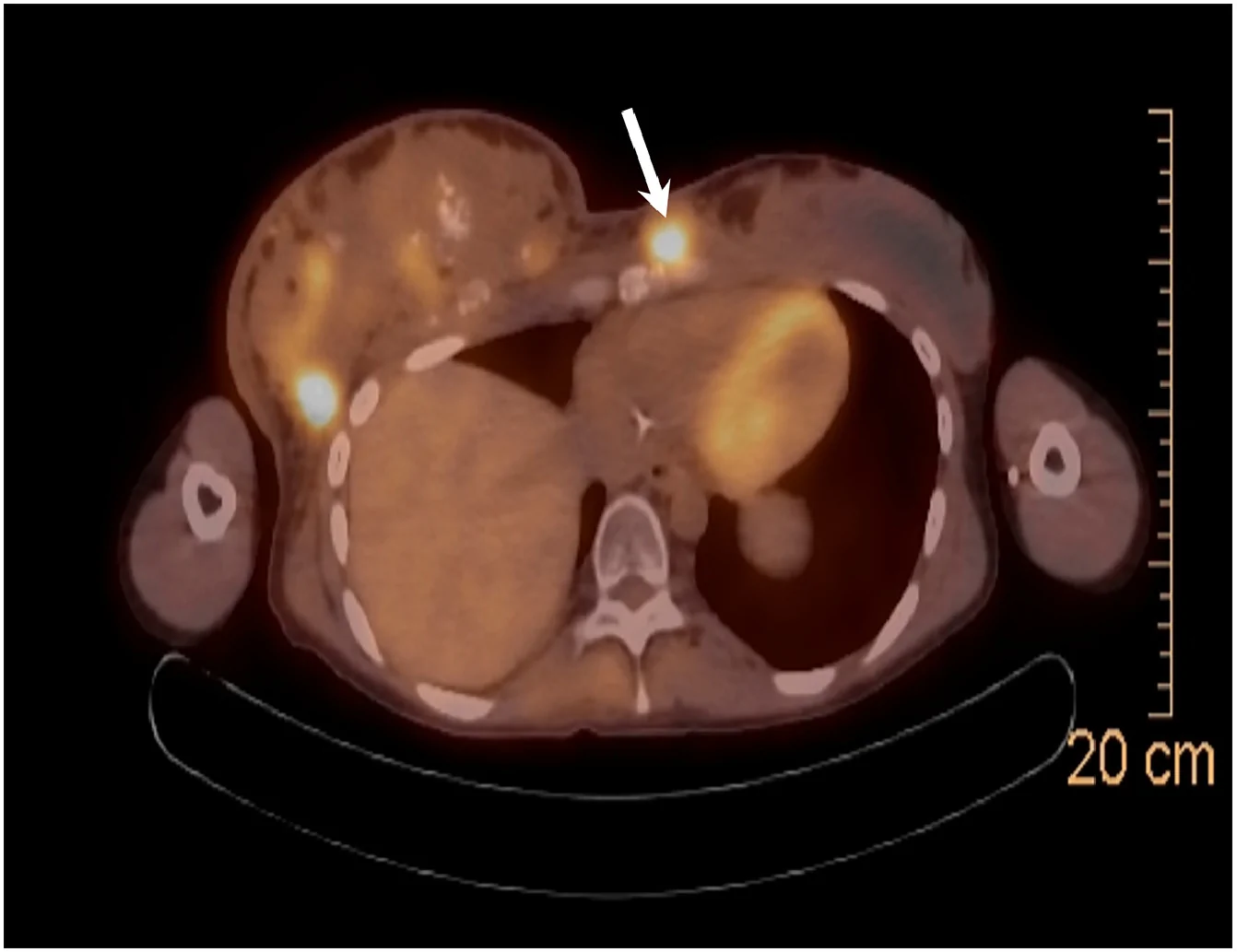
PET/CT obtained 6 months after the prior PET/CT shows decreased size and metabolic activity of the right breast mass, consistent with positive treatment response to chemoradiation. However, there is a new FDG-avid mass in the inner aspect of the left breast, consistent with malignancy (arrow). The lungs are incompletely imaged.

### Complications and outcome

Approximately 3 years after initial presentation, imaging demonstrated tumor necrosis, chest wall ulceration, a right hydropneumothorax, and new lung opacities likely representing infection (Fig. 6). Following palliative mastectomy, her course was complicated by respiratory failure requiring intubation. Final chest radiography showed hydropneumothorax and diffuse bilateral airspace opacities (Fig. 7). Despite maximal support, she developed septic shock and died shortly thereafter, approximately 3 years after initial presentation, from acute respiratory distress syndrome (ARDS) secondary to metastatic Ewing sarcoma.

**Fig. 6:**
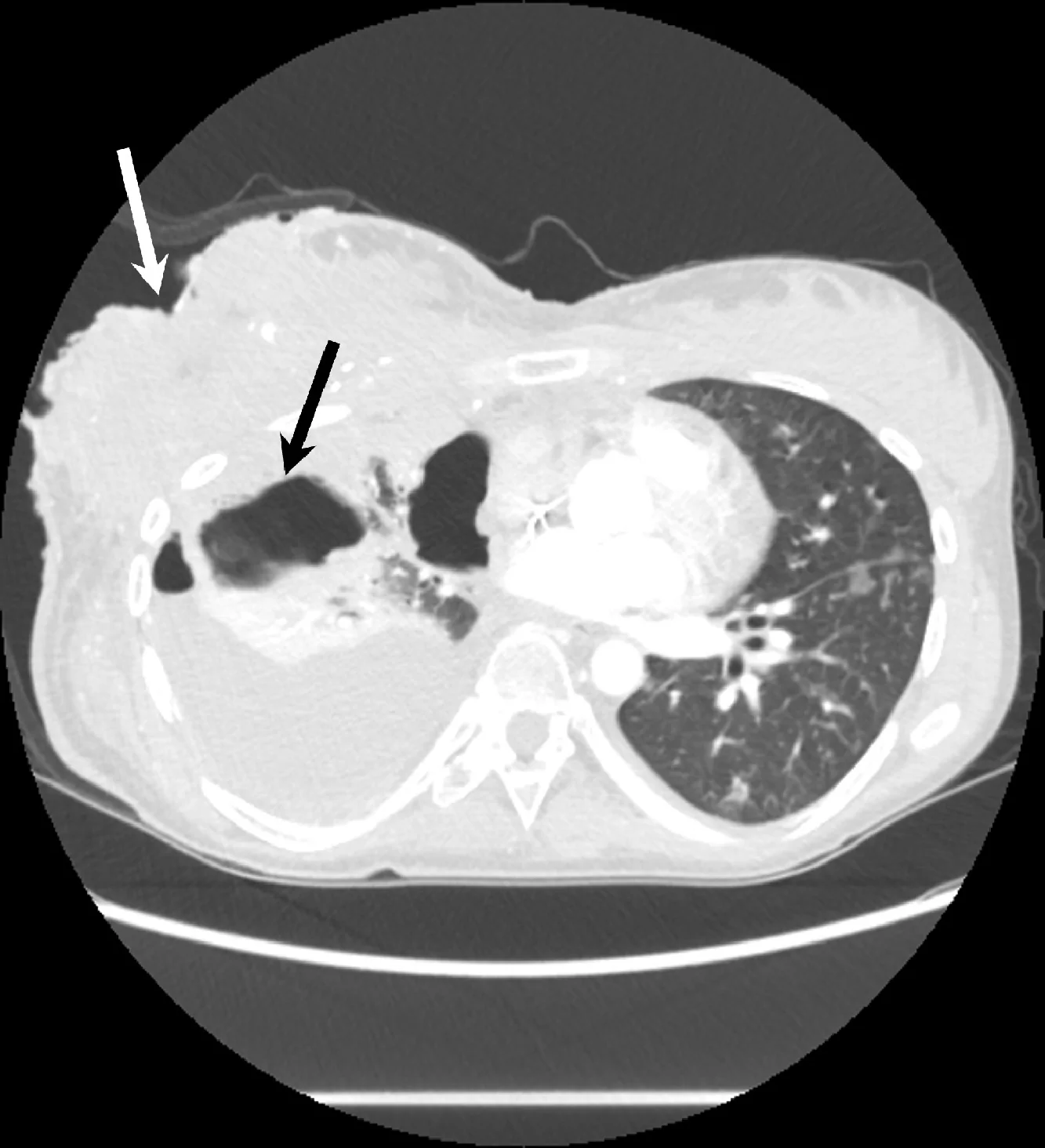
Axial chest CT image (lung window) shows a right chest wall mass with new ulceration (white arrow). The pulmonary component of the lesion appears smaller but irregular. There is a right hydropneumothorax with a new pneumothorax component (black arrow), likely related to progressive tumor necrosis and disruption of the adjacent pleural space. There are new nodular ground glass opacities in the left lung, favored to be infectious. No pleural catheter was present at this time.

**Fig. 7:**
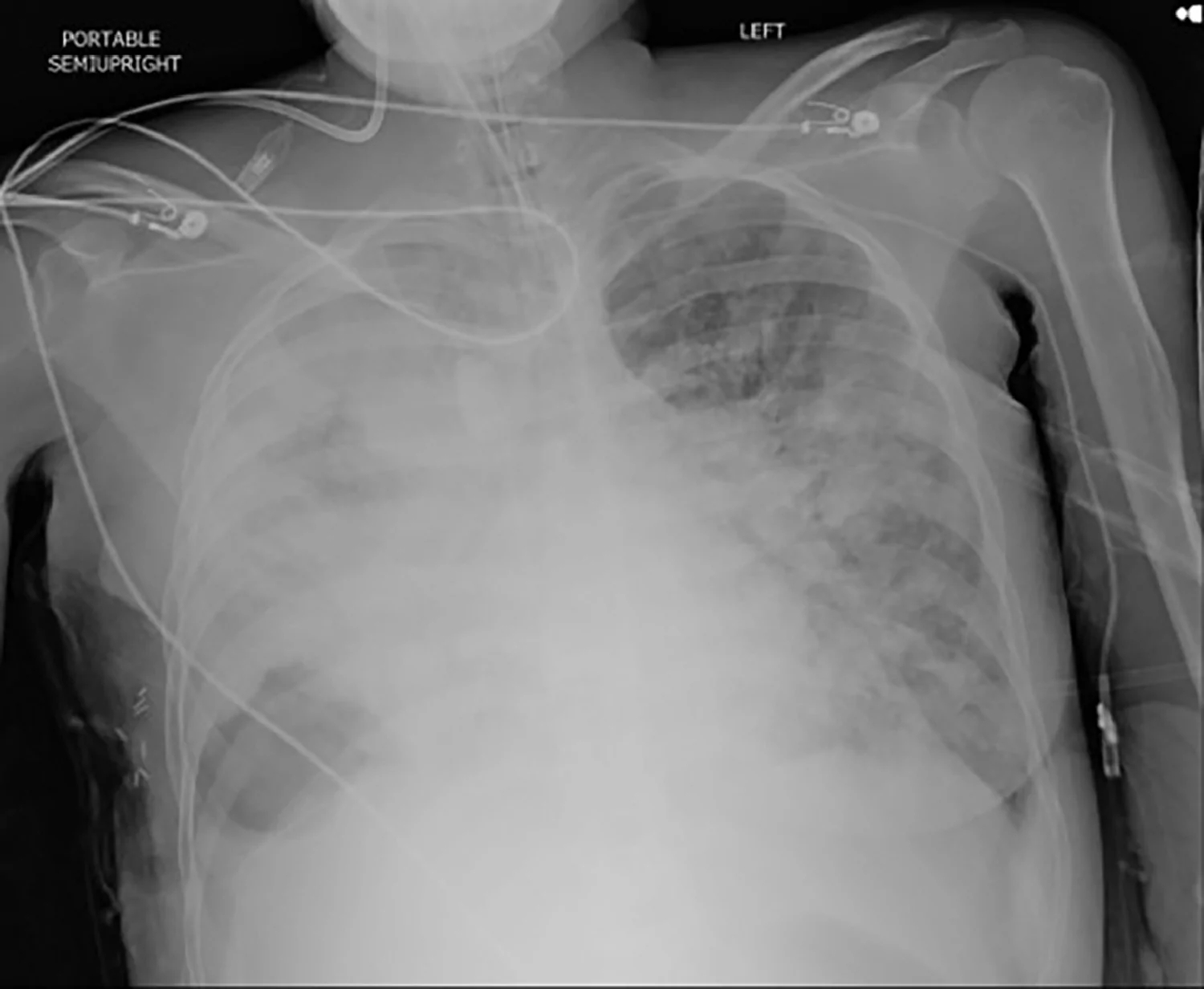
Portable chest radiograph shows endotracheal tube, feeding tube, left upper extremity peripherally inserted central catheter (PICC), and right PleurX catheter. The patient is status post right mastectomy with associated surgical clips. There is a right hydropneumothorax, with pneumothorax visible at the right lung base. There are diffuse bilateral airspace opacities which may represent infection, edema, and/or ARDS. The patient was extubated and died 12 hours after this chest radiograph.

## Discussion

Ewing sarcoma is an aggressive malignancy that most commonly arises in the long bones and pelvis, and while rare overall, primary involvement of the chest wall represents the most common malignant chest wall tumor in pediatric and young adult populations [4]. In the presented case, the exceptionally large size of the chest wall tumor at the time of definitive treatment created diagnostic uncertainty regarding its precise site of origin. While imaging favored a rib origin, involvement of the adjacent breast tissue raised consideration of a primary extraosseous Ewing sarcoma of the breast, an exceedingly rare origin described only in isolated case reports. This distinction is clinically meaningful, as breast-origin Ewing sarcoma carries among the poorest prognoses of all breast cancer subtypes, further highlighting the importance of accurate site localization when breast tissue is involved [1,4].

The atypical location along the anterior chest wall likely contributed to delayed presentation and diagnostic ambiguity. Symptomatically, tumors abutting breast tissue may mimic benign musculoskeletal or breast pathology, often presenting with regional pain and swelling rather than systemic symptoms that prompt early evaluation. A study of 26 patients under 30 years of age with rib-based Ewing sarcoma found that the most common presenting features were pain (23/26 patients), a palpable mass (11/26 patients), and pleural effusion on imaging (10/15 imaged), while a malignancy was suspected at initial presentation in only 5 cases and identified on initial radiography in only 4 of 15 cases. Notably, 12 of 26 patients ultimately died from the disease with a median survival of 2.4 years, highlighting both the diagnostic challenge and the consequence of delayed recognition in this younger population with less overt clinical presentations of Ewing sarcoma [7].

In this patient, delayed presentation and loss to follow-up allowed for continued tumor growth and progression to macrometastatic disease. Tumor size is a well-established adverse prognostic factor in Ewing sarcoma, with larger tumor volume associated with significantly decreased event-free survival. Ewing sarcoma metastasizes hematogenously, most commonly to the lungs, bone, and bone marrow, and the presence of overt metastases is associated with markedly worse outcomes [8]. The substantial tumor burden in this case likely contributed not only to local invasion of breast tissue but also to the pulmonary and osseous involvement, leading to pleural effusion and vertebral compression fractures. The development of hydropneumothorax occurred in the setting of extensive tumor necrosis and chest wall ulceration, suggesting disruption of the adjacent pleural space as a likely mechanism. The patient’s course was ultimately complicated by ARDS and death following a palliative mastectomy.

The current standard of care for Ewing sarcoma emphasizes a multimodal approach incorporating systemic chemotherapy, surgery, and/or radiation therapy. Neoadjuvant chemotherapy is critical both for tumor volume reduction and for treatment of presumed micrometastatic disease, as Ewing sarcoma is widely considered to harbor *a priori* micrometastases at diagnosis [9,10]. Without systemic therapy, survival is approximately 10%, underscoring the necessity of prompt treatment initiation [1]. Delays in diagnosis and care represent potentially modifiable factors with significant prognostic implications. Initiation of therapy within 28 days of diagnostic biopsy has been associated with significantly improved survival outcomes in adults and children with Ewing sarcoma [11]. In this case, interruptions in care likely limited therapeutic options and contributed to disease progression and spread. Maintaining a high index of suspicion for atypical presentations of Ewing sarcoma is essential to facilitate early imaging, prompt diagnosis, and timely initiation of multimodal therapy, all of which remain key determinants of patient outcomes.

## Conclusion

This case illustrates the potential for chest wall Ewing sarcoma to reach an exceptionally large size and progress to multi-organ metastatic disease. Given its large size, it is unclear if this sarcoma originated from the right breast or from a right anterior rib, although the latter is favored. Although rare, the aggressive metastatic nature of Ewing sarcoma of the chest wall, particularly in young patients with delayed presentation and treatment, underscores the need for increased awareness of the clinical and imaging findings of the disease.

## Patient consent

Informed consent for this case was obtained from the patient’s next of kin.
